# Placebo-Related Adverse Events in Rheumatoid Arthritis

**DOI:** 10.3390/biom12020303

**Published:** 2022-02-14

**Authors:** Ratna Shree Sharma, Johannes Pallua, Michael Schirmer

**Affiliations:** 1 Rheumatology Research Group, Department of Internal Medicine, Clinic II, Medical University of Innsbruck, Anichstraße 35, 6020 Innsbruck, Austria; ratna.shree.sharma@gmail.com; 2 University Hospital for Orthopedics and Traumatology, Medical University of Innsbruck, Anichstraße 35, 6020 Innsbruck, Austria; johannes.pallua@i-med.ac.at

**Keywords:** adverse effects, nocebo effect, placebo-controlled trial, complementary medicine, shared decision-making, musculoskeletal diseases, rheumatology

## Abstract

Prospective, double-blind, randomized, placebo-controlled studies are considered to provide the highest quality of interventional evidence. This meta-analysis summarizes the frequencies of adverse events according to the Medical Dictionary for Regulatory Activities (MedDRA) in the placebo arms of 101 such studies in rheumatoid arthritis, including a total of 17,150 patients in the placebo arms and 37,819 patients in the verum arms. Placebo-treated patients reported more than one adverse event in a median of 55.0%, 65.5%, and 72.5% (compared to 72.3% in the verum arms), and a serious adverse event in 2.5%, 5.8%, and 8.6% (compared to 5.9% in the verum arms), with stable doses of corticosteroids, conventional synthetic disease-modifying antirheumatic drugs (DMARDs), and biological DMARDs as background therapies, respectively. Odds ratios were comparable between placebo and verum arms for nausea (1.00 with 95% confidence interval (CI) 0.86–1.17), for hepatobiliary disorders (1.08 with CI 0.85–1.36), for abnormal hepatic functions (1.09 with CI 0.83–1.44), and general disorders and administration site conditions (1.39 with CI 0.95–2.03). A publication bias has to be assumed for nausea (*p* = 0.018; Egger’s test), diarrhoea (*p* = 0.022), and serious infections and infestations (*p* = 0.009). In conclusion, patients should be aware that “adverse events” may occur even with placebo medication, independent from an additional verum medication added to the background therapy. Further studies are warranted to respect and overcome the psychological and other issues related to these placebo-related “adverse events”.

## 1. Introduction

Rheumatoid arthritis (RA) affects about 0.5% of the population in the industrialized world [1]. According to the treat-to-target initiative, RA patients should rapidly achieve remission or at least low disease activity [2], as otherwise joint destruction leading to severe disability together with premature mortality may occur [3]. Additionally, there is high agreement between the experts of this treat-to-target initiative that treatment must be based on a shared decision between the patient and rheumatologist [2]. With shared decision-making, physicians and informed patients work together to understand the patient’s situation better, and determine how best to address it [4]. From the patients’ perspective, knowledge about the disease itself and available treatment options must be considered a prerequisite for shared decision-making [5]. For this purpose, information on the pros and cons of the available options is usually derived from placebo-controlled, double-blind, randomized trials, which provide high-level evidence for the conversation between patients and their clinicians. However, such high-level evidence is often not available for alternative approaches, although complementary and alternative medicine is commonly used (e.g., in 37.9% of Australians with inflammatory arthritis) [6] and may even lead to delayed initiation of treatment with disease-modifying antirheumatic drugs (DMARDs) [7]. Missing information may thus lead to underestimation of adverse events in complementary medicine compared to recommended treatment strategies.

Knowledge on possible adverse events of placebos could be helpful to define a level of minimal information on potential adverse events. In 1964, Arthur K Shapiro proposed a definition of placebo as any therapeutic procedure or its component which happens to influence symptoms, syndromes, diseases, or patients—but without any expected effect on the specific condition [8]. Today, the placebo effect is defined as a beneficial health outcome unrelated to the direct effects of what is carried out or given but is achieved by the same inert substance [9]. On the contrary, the term “nocebo” was introduced into the literature to describe the “negative” placebo effects and to differentiate between adverse and beneficial effects [10]. Two types of nocebo effects were proposed, featuring the primary nocebo effect with reduced overall treatment efficacy and the nocebo or adverse effect going along with unpleasant adverse events [11]. For example, in patients who had discontinued statin therapy because of side effects, 90% of the adverse events elicited by a statin challenge were also elicited by placebo [12].

Today, placebos are regularly used as controls in prospective, double-blind trials, with randomization of participants before study entry into a verum and a placebo arm to eliminate any treatment-related bias. These studies are considered the most reliable source of clinical evidence, they were used to analyze the frequency of adverse effects in placebo-treated patients. Besides the high frequency of such adverse events even in placebo-treated RA patients, the underlying reasons for a possible influence of the verum medication on adverse events in randomized studies deserve further investigation.

## 2. Materials and Methods

This systematic literature review aims to carry out the meta-analysis of placebo-related adverse events in double-blind, randomized controlled trials. The literature screening and evaluations were carried out by R.S.S. in accordance with guidelines set up together with M.S. The PRISMA-P2020 checklist was applied [13].

### 2.1. Selection of Publications

Literature was searched in PubMed and the Clinical Trials registry [14], published between 1969 and May 2020. Items for the PubMed search were (“placebo”[All Fields]) AND (“rheumatoid arthritis”[All Fields]) AND (“adverse effects”[All Fields]). The items for ClinicalTrials registry search were “Completed”, “Studies with Results”, “Interventional Studies”, “Rheumatoid Arthritis”, “placebo”, “Adult, Older Adult”, and “Phase 3, 4” (searched on 12 June 2020 in PubMed and on 17 June 2020 in the ClinicalTrials registry).

All studies published in the English language were included if they were (1) randomized, double-blind clinical trials, (2) phase III or phase IV, (3) with adult patients over the age of 18 years, and (4) with results available from the completed placebo-controlled phase. Studies were excluded if they were (1) terminated before planned completion, (2) with corticosteroids as only verum, (3) with investigational drugs as verum but without approval by authorities, or (4) non-pharmacological studies. A duplicate search was performed using the Mendeley software (Mendeley Ltd., London, UK) version 1.19.8. Studies were then screened for availability, relevant contents, and reporting of adverse events. Additionally, data from 8 parallel groups with different verum were excluded for further analyses. 

### 2.2. Data Collection with the Coding of Adverse Events

After the selection of appropriate studies, patient and disease characteristics at the time of randomization were manually extracted as baseline data. Then, data on clinical outcome, adverse events, percentage of people withdrawn due to adverse events, and mortality data were collected for both the verum- and the placebo-groups. In studies with several dosages of verum, the dosages and other data were averaged as verum data. For collecting data on adverse events, the Medical Dictionary for Regulatory Activities (= MedDRA), a multi-axial, multi-level hierarchical terminology, was used as applied to code adverse events in more recent studies. The five levels of MedDRA hierarchy include (1) a total of 26 System Organ Classes (=SOCs), (2) 337 high-level group terms, (3) 1737 high-level terms, (4) 24,289 preferred terms, and (5) 81,812 lowest level terms [15]. This coding terminology allows the combination of adverse data in case of infrequent reporting. If not already listed in the publications, the adverse events were assigned to the MedDRA terms to provide MedDRA-compatible data.

### 2.3. Data Preparation and Statistics

Studies were grouped into those with nonsteroidal anti-inflammatory drugs (NSAIDs), conventional synthetic (cs) DMARDs, targeted synthetic (ts) DMARDs, and biological (b) DMARDs as verum. Within these groups, studies were sub-grouped according to their mechanism of action, with dihydroorotate dehydrogenase (DHODH) inhibitor leflunomide and methotrexate as csDMARDs, januse kinase (JAK) inhibitors as tsDMARDs and blockers of antitumor necrosis factor-alpha (αTNFα), cytotoxic T lymphocyte-associated protein-4 (αCTLA4), the B-cell marker CD20 (αCD20) or the interleukin-6 receptor (αIL6R) and the interleukin-1 receptor antagonist (IL1RA) as bDMARDs. In the case of 2 different verum groups, data of the placebo arm were used for comparisons with both verum groups separately. Since many years, placebo-groups without any DMARD such as methotrexate have not been performed.

The following SOCs had available data in less than 10 studies (out of 101 included studies): immune system disorder; endocrine disorder; eye disorders; ear and labyrinth disorders; cardiac disorders; pregnancy, puerperium, and perinatal conditions; reproductive system and breast disorders; congenital, familial and genetic disorders; and surgical and medical procedures and social circumstances, and were excluded from further analyses.

Statistical analyses were performed using the IBM SPSS Statistics software program (version 27, IBM Corporation, Armonk, NY, USA) and MedCalc (version 19.8, MedCalc Software, Ostend, Belgium). Data were weighted by the total number of patients in the respective study arms. Descriptive statistics included medians and a range of data (minimum and maximum). Further analyses included odds ratio (OR) and 95% confidence interval (CI) for OR. With a Pearson’s correlation coefficient (r) > +0.5 the tested correlations were considered as strong (between verum and placebo groups). Inconsistency (I^2^) was calculated to choose the *p*-value for ORs from a fixed-effect model or a random-effect model. In case of homogeneous studies (with I^2^ ≤ 50%), the *p*-value for ORs from a fixed-effect model of meta-analysis is used, while in case of heterogeneous studies (with I^2^ > 50%), a random-effect model is used. *p*-values of Egger’s test were used to assess a possible publication bias (with *p* > 0.05 in case of no publication bias). Diagrams of forest plots and funnel plots were designed using the free trial version of MedCalc for Windows.

## 3. Results

**Figure 1:**
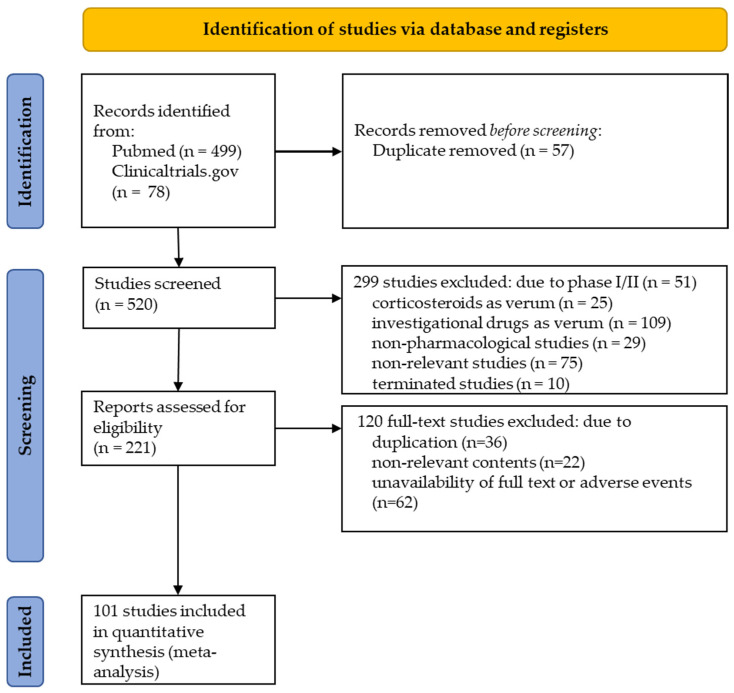
Flow diagram of record selection according to the PRISMA-P strategy.

A total of 101 placebo-controlled, double-blind, randomized studies were identified listing adverse events in placebo-exposed patients with RA (with references listed in the Supplementary Material). The flow diagram for the PRISMA-P strategy with the selection process out of the initial 520 studies is shown in Figure 1, excluding 57 duplicates, 299 inadequate studies according to title and abstract, and 120 studies that did not meet other inclusion criteria. Studies were grouped according to the verum medications with bDMARDs (*n* = 78), tsDMARDs (*n* = 14), csDMARDs (*n* = 3), and NSAIDs (*n* = 6). bDMARDs included blockers of αTNFα (*n* = 43), αIL6R (*n* = 15), αCTLA4 (*n* = 10) or αCD20 and IL1RA (each with *n* = 5). Follow-up of placebo-controlled studies was a median of 24 (2–104) weeks.

Background therapies were stable doses of corticosteroids only (in 11 studies with 1861 patients in the placebo arms), a csDMARD (in 88 studies with 14,879 patients), or a bDMARD (in 2 studies with 410 patients).

### 3.1. Demographics and Disease Characteristics of Patients in Placebo-Groups

A total number of 17,150 RA patients were treated with placebos in the 101 double-blind and randomized interventional studies, as controls for another 37,819 patients in the verum arms of these studies. Details on the placebo patients’ demographics are given in Table 1.

In all the placebo arms, a median of 79.7% (53.4–89.0%) was female, the median age at disease onset was 52.3 (46.7–62.7) years, and the median disease duration at the time of study entry was 8.4 (0.3–14.0) years. Rheumatoid factor was positive in 80.0% (56.8–97.0%) and anti-citrullinated peptide antibodies were positive in 82.0% (56.0–100.0%) of placebo patients. With these comparable data of RA and patients’ characteristics in mind, further analyses were performed.

### 3.2. Frequency of Adverse Events in Placebo-Treated Patients

At least one adverse event occurs in a median of more than 55.0% of placebo-treated patients with stable doses of corticosteroids, sometimes even with serious adverse events or withdrawal from the study due to an adverse event (Table 2).

Overall, placebo patients with csDMARDs or bDMARDs as background treatments usually report more adverse events than patients with stable doses of corticosteroids, which can be explained by the fact that that csDMARDs, such as MTX and leflunomide, may cause nausea and hepatobiliary disorder, and bDMARDs are related to more infections. Therefore, in Table 3 and Table 4 adverse events are listed according to the background treatment both in the placebo group and the serum group.

In detail, MedDRA-coded adverse events are listed in Table 3. Especially gastrointestinal disorders, as well as infections and infestations, occur in an averaged median of 16.2% and 19.9% of all placebo patients with stable doses of corticosteroids as the background therapy, respectively, and even more often in patients with a csDMARD as the background therapy. Sinusitis is more frequently reported in placebo patients treated with stable doses of corticosteroids as background therapy than in those placebo patients with csDMARDs or bDMARDs as background treatments, and even in verum-treated patients (13.0% vs. 3.9%, 3.8% and 6.4%, respectively).

### 3.3. Comparisons of Adverse Events between Placebo and Verum Patients

The following analyses are based on data already summarized in Table 3. Using odds ratios (OR) with 95% confidence intervals (95% CI) to compare verum and placebo patients, revealed significant ORs for more than one adverse event (OR = 1.31 [95% CI 1.21–1.42], *p* = 0.000), a severe adverse event (OR = 1.17 [95% CI 1.08–1.27], *p* = 0.000), and study withdrawal due to an adverse event (OR = 1.39 [95% CI 1.19–1.61], *p* = 0.000).

The question arises as to whether the prevalence in the placebo patients correlate with those in the verum patients. With a Pearson’s correlation coefficient (r) of 0.921 and *p* = 0.000, a strong positive correlation was found between verum- and placebo groups for at least 1 adverse event. Additionally, for serious adverse events (r = 0.822; *p* = 0.000) and for withdrawal from the study due to an adverse event (r = 0.664; *p* = 0.000), there were strong positive correlations between verum- and placebo-treated patients. 

**Figure 2:**
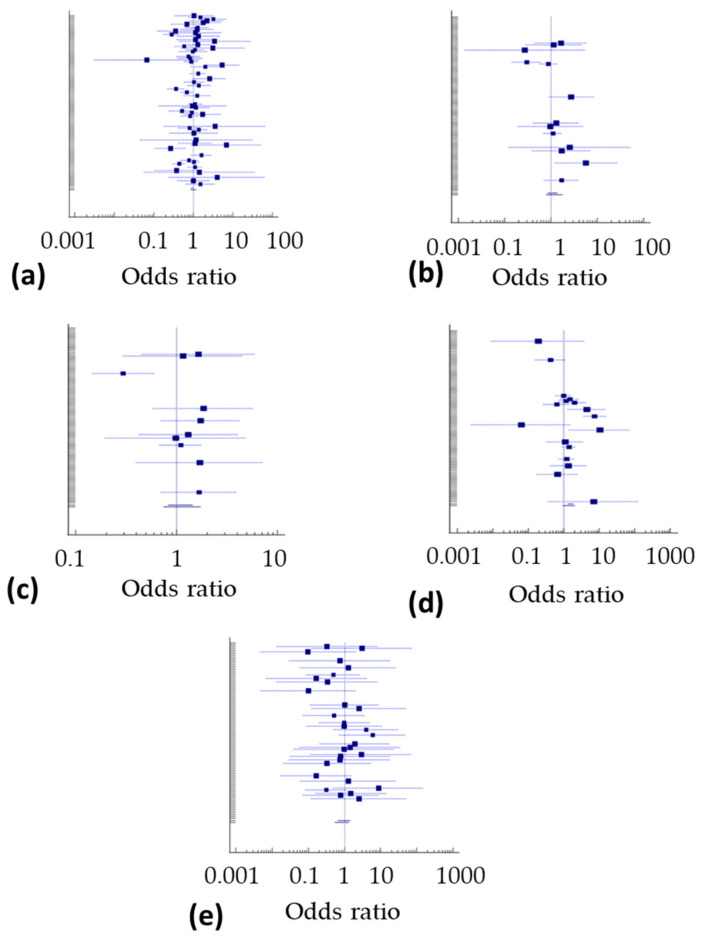
Forest plot summarizing odds ratios for (**a**) nausea; (**b**) hepatobiliary disorders; (**c**) abnormal hepatic functions; (**d**) general disorders and administration site condition; and (**e**) death with AE. The frequencies of these adverse events were not affected by verum medication compared to the placebo medication.

Calculation of ORs further showed that at least some gastrointestinal disorders and general disorders occur in both placebo and verum groups without a different prevalence, independent from the therapeutic background (stable doses of corticosteroids, csDMARDs or bDMARDs) (Table 4). In detail, for nausea OR was 1.00 (95% CI 0.86–1.17; *p* = 0.985, for hepatobiliary disorders OR was 1.08 (95% CI 0.85–1.36; *p* = 0.542) and for abnormal hepatic functions OR was 1.09 (95% CI 0.83–1.44; *p* = 0.537), as for general disorders and administration site conditions OR was 1.39 (95% CI 0.95–2.03; *p* = 0.086) when comparing placebo and verum patients. To illustrate this observation, Forest plots are depicted in Figure 2, summarizing these results from the interventional studies.

**Figure 3:**
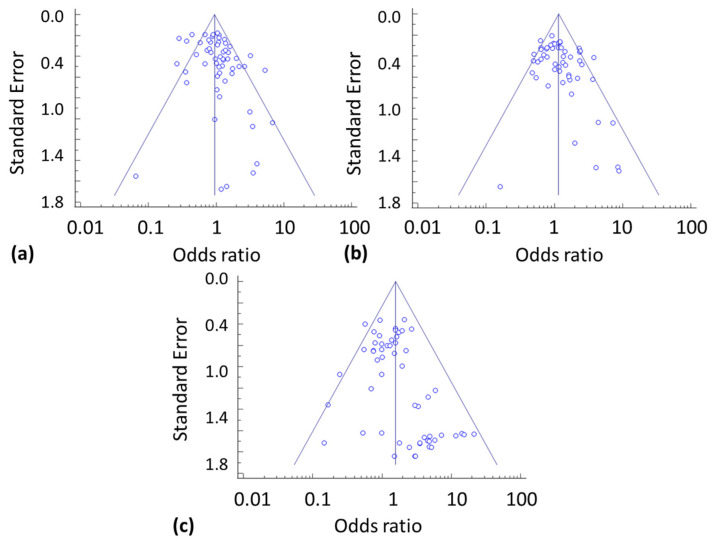
Funnel plots on the reporting of selected adverse events in placebo groups, showing the effect size (odds ratio) as a function of included studies (natural log of standard error). A publication bias has to be suggested for (**a**) nausea but not for (**b**) diarrhoea and (**c**) serious infections and infestations.

Using the Egger’s test, a publication bias has to be assumed for nausea with *p* = 0.018, diarrhoea with *p* = 0.022, and serious infections and infestations with *p* = 0.009 (Table 4). Therefore, Funnel plots have been performed, which can also identify a publication bias in interventional studies. The Funnel plots show that most dots are drawn towards one side and dots are not distributed symmetrically on both sides (Figure 3).

## 4. Discussion

This meta-analysis analyzed 101 double-blind, randomized interventional trials, and describes adverse events in more than 55% of placebo-treated RA patients. This perspective of adverse events observed even in the placebo arms of RA studies has not been analyzed before.

We performed this meta-analysis, as some RA patients are threatened by the list of possible adverse events they are informed about before taking a verum medication. However, as more than 55% of the placebo-treated RA patients were also affected by one or more adverse events, the natural course of disease and placebo-related effects should not be underestimated. In total, 88 out of the 101 studies had csDMARDs as the background therapy, thus allowing a perfect analysis of the placebo group. Additionally, a comparison between the different placebo groups with stable doses of corticosteroids, csDMARDs or bDMARDs as background therapies is possible (with adverse events in 65.5% vs. 72.3% and serious adverse events in 5.8% vs. 5.9% of placebo and verum arms, respectively). Most interestingly, even severe adverse events were reported by 2.5% of placebo-treated patients under stable corticosteroids as the background therapy, and 4.4% of these placebo-treated RA patients withdrew from the studies due to an adverse event. These data were even higher in patients with csDMARD or bDMARD as background treatments (Table 2). It can be assumed that at least the data from patients with stable doses of corticosteroids as the background therapy provide the most reliable data available for reported adverse events in placebo-treated RA patients, as placebo groups without any background therapy will not be considered as ethical in the future [16]. These data are comparable to data from patients with brain diseases, with rates of adverse events in the placebo groups varying between 25% in symptomatic treatment for multiple sclerosis and almost 80% in motor neuron disease, and rates of withdrawal from studies due to an adverse event varying between 2% in multiple sclerosis and nearly 10% in Parkinson’s disease [17].

Because of the different types of background treatments, it is difficult to predict the absolute number of adverse events for a new DMARD. However, usually, patients have been treated with the background therapy over several weeks before entry into the study, thus ensuring tolerability of the background therapy over time. Nevertheless, the observation that patients with stable doses of corticosteroids report less adverse events than those with csDMARDs or bDMARDs, can be explained by the fact that csDMARDs, such as MTX and leflunomide, may cause nausea and hepatobiliary disorders, and bDMARDs are related to more infections. Therefore, in Table 3 and Table 4, adverse events are listed according to the background treatment both in the placebo group and the verum group, to allow a better comparison. Still, negative synergistic effects of the background therapy with the new DMARD may occur, as infections and infestations can be expected in 19.9% of placebo-treated patients with stable doses of corticosteroids and 31.4% with the background of csDMARDs (Table 3). Therefore, with 37.0%, an increased prevalence of infections and infestations in the verum arms could be interpreted as a calculated prevalence of only 5.6% to be attributed to the verum agent. Such risk calculations would influence both patients’ and their clinicians’ attitudes when choosing between additional or alternative treatment options, although patients still need appropriate information to search for early diagnosis and treatment of suspected infections and infestations.

There is a great level of agreement amongst experts about the need of shared decision-making between patients and their rheumatologists [2]. RA patients need to be informed about the adverse events of the verum medications, but data on possible adverse events in placebo patients may add a new perspective on the frequency of these adverse events. Until today, patients may not be aware that even placebo treatment can lead to one or more, sometimes even severe adverse events, as shown in this meta-analysis. Data from such a meta-analysis of 101 double-blind, randomized controlled studies with a total of 17.150 placebo-treated patients can be considered as high-quality data and may provide additional information for clinicians when discussing possible adverse events with their patients. This information may become relevant especially for patients tending to avoid verum medication because of possible adverse events, with the consequence of subsequent low compliance if they are not convinced about the low risk–benefit ratio of the new verum medication.

Without a doubt, patients with more than one adverse event, a severe adverse event, and study withdrawal due to an adverse event are more often reported with verum than placebo treatment. The prevalence of these adverse events strongly correlates between placebo- and verum-treated patients, as already shown for an extensive range of medical conditions [18]. However, in RA patients, the prevalence rates of adverse events are at least sometimes comparable between placebo-and verum-treated patients, as for nausea, hepatobiliary disorders, abnormal hepatic functions, general disorders, and administration site conditions (Figure 2 and Table 3). It appears that all patients have been equally affected by these adverse events. However, according to the Egger’s test analysis together with a Funnel plot (Table 4 and Figure 3), a publication bias has been identified for nausea, which is a non-laboratory, subjective adverse event, but not in other more objective adverse events, which may be a result of patients’ information before study initiation. As almost every study permitted the additional use of analgesics, nonsteroidal anti-inflammatory drugs, and oral corticosteroids, one can only speculate about their role in the occurrence of gastrointestinal adverse events.

This meta-analysis has several important limitations because of its retrospective design, based on studies’ reports from literature and registries instead of individual patients’ data. Adverse events were searched for in only two data banks, which were considered as the most reliable to report adverse events. For sure, insights into the individual patients’ data from the clinical studies would allow more detailed analyses. For example, the sex difference of reporting adverse events in placebo-treated RA patients was not examined, although such differences had been reported in neuropathic pain trials [19] probably caused by stress, anxiety, and the endogenous opioid system [20]. 

MedDRA, as a highly specific standardized medical terminology to facilitate the sharing of regulatory information, was developed in the late 1990s. Therefore, older studies did not use the MedDRA nomenclature, and published only the most frequent adverse events with the possible consequence of providing incomplete reports for rare adverse events. Therefore, we omitted all adverse events occurring in less than 10% of the studies. Additionally, SOCs were excluded when data were available from less than 10 studies. The influence of background therapies has been discussed earlier. Of note, background therapy is usually kept stable over weeks before recruitment into a study as a prerequisite of study entry, so that adverse events of the background therapy may occur rather before than during the study. Data on the length of background therapy kept stable before study entry; however, were not analyzed. As almost every study permitted further use of analgesics, NSAIDs, and oral corticosteroids, these medications might have influenced adverse events in placebo-treated patients more than in the verum-treated patients. However, the doses of these medications were not always available and therefore not analyzed.

As the studies differed in patient size, the meta-analysis weighted the data according to the total number of patients using the weight cases option of the SPPS software. Using the Egger’s test analysis and the Funnel plot, despite all available peer-reviewed study reports, a publication bias became evident for nausea and diarrhoea as adverse events.

## 5. Conclusions

This meta-analysis of double-blind, randomized, and placebo-controlled interventional studies reports adverse events in 55.0%, 65.5%, and 72.5% of placebo-treated RA patients (compared to 61.0%, 73.1%, and 74.8% of verum-treated patients) with stable doses of corticosteroids, csDMARDS and bDMARDs as background therapies, respectively. Therefore, patients should be aware that “adverse events” may occur independent from an additional verum medication added to the background therapy. Further studies are warranted to analyze the issues underlying these placebo-related “adverse events”.

## Figures and Tables

**Table 1:** Patients’ demographics and disease characteristics of placebo groups according to the study of verum medication.

DMARD of Verum Group	Female (%)	Age at RA Onset (Years)	RA Duration (Years)	RF+ (%)	ACPA+ (%)
NSAIDs (n = 1369)	80.6 (73.0–85.2)	53.8 (51.0–59.2)	9.9 (4.2–11.0)	50.3	NA
csDMARDs (n = 460)	78.6 (74.0–80.5)	55.4 (53.6–56.6)	8.3 (6.1–12.7)	82.8 (78.0–87.6)	NA
tsDMARDs (n = 2504)	78.0 (70.0–86.1)	52.8 (47.7–56.3)	8.6 (1.3–14.0)	74.0 (56.8–97.0)	75.8 (56.8–92.0)
bDMARDs (n = 12,817)	79.2 (66.0–88.5)	49.6 (47.7–54.7)	8.1 (0.5–11.4)	84.4 (72.9–96.8)	79.9 (73.9–85.8)
αCTLA4 (n = 1220)	79.2 (66.0–88.5)	49.6 (47.7–54.7)	8.1 (0.5–11.4)	84.4 (72.9–96.8)	79.9 (73.9–85.8)
αCD20 (n = 853)	79.0 (76.2–85.5)	52.1 (48.1–54.0)	7.5 (0.9–11.7)	77.5 (75.0–87.0)	78.0
IL1RA (n = 1012)	74.6 (70.2–85.1)	54.5 (52.2–57.0)	10.0 (3.7–10.7)	74.3 (69.4–78.0)	NA
αIL6R (n = 3608)	80.7 (64.0–87.5)	52.9 (49.6–55.8)	9.0 (0.4–12.3)	79.4 (50.0–89.0)	83.6 (78.0–86.0)
αTNFα (n = 6124)	79.8 (53.4–89.0)	52.0 (46.7–62.7)	7.4 (0.3–14.0)	83.3 (67.0–100.0)	80.8 (56.0–100.0)

Data are weighted according to the total number of placebo patients in each study and given as median (minimum–maximum). ACPA, anti-citrullinated peptide antibody; αCTLA4, anti-cytotoxic T-lymphocyte-associated protein-4; IL1RA, interleukin-1 receptor antagonist; αIL6R, anti-interleukin-6 receptor; NA, data not available; RF, rheumatoid factor; and αTNFα, antitumor necrosis factor-alpha.

**Table 2:** Summary of adverse events in percentages of placebo patients compared to verum-treated patients depending on background therapy.

Background Therapy	Placebo Patients [%] (n = 17,150)			Verum Patients [%] (n = 37,819)		
	Stable Doses of Corticosteroids (n = 1861)	csDMARDs (n = 14,879)	bDMARDs (n = 410)	Stable Doses of Corticosteroids (n = 3633)	csDMARDs (n = 33,649)	bDMARDs (n = 537)
≥1 adverse event	55.0 (16.0–81.6)	65.5 (10.5–98.1)	72.5 (71.4–72.5)	61.0 (15.7–94.6)	73.1 (15.6–98.8)	74.8 (70.0–79.5)
Serious adverse event	2.5 (0.0–5.7)	5.8 (0.0–33.8)	8.6 (5.8–11.3)	2.0 (0.0–27.5)	5.7 (0.0–29.3)	8.3 (6.1–10.5)
Study withdrawal due to adverse event	4.4 (1.8–20.0)	3.7 (0.0–36.0)	14.1 (2.5–25.6)	4.5 (0.0–17.0)	5.0 (0.0–46.0)	8.6 (3.6–13.6)
Adverse event with death	0.0	0.0 (0.0–5.0)	0.4 (0.0–0.7)	0.0 (0.0–0.7)	0.1 (0.0–3.0)	0.1 (0.0–0.3)

Values are given in median percentages (minimum–maximum). AE, adverse events; DMARD, disease-modifying antirheumatic drug; csDMARD, conventional synthetic DMARD; bDMARD, biological DMARD.

**Table 3:** MedDRA list of adverse events in percentages of placebo patients compared to the verum-treated patients depending on the background therapy (in alphabetical order, data are listed only with adverse events occurring more frequently than in 5% of the patients, at least in one group, and ranges are given only in groups with data available from more than one study).

Background Therapy	Placebo Patients [%] (n = 17,150)			Verum Patients [%] (n = 37,819)		
	Stable Doses of Corticosteroids (n = 1861)	csDMARDs (n = 14,879)	bDMARDs (n = 410)	Stable Doses of Corticosteroids (n = 3633)	csDMARDs (n = 33,649)	bDMARDs (n = 537)
Blood and lymphatic system disorder	1.5 (1.4–1.6)	2.5 (0.0–10.0)	2.7 (2.3–5.1)	2.9 (1.4–5.0)	4.2 (0.0–29.0)	4.2 (1.2–7.2)
- Neutropenia	0.0	0.2 (0.0–4.0)	5.1	NA	8.3 (0.7–29.0)	5.4
Gastrointestinal disorders	16.2 (0.6–23.2)	14.0 (5.6–21.4)	NA	19.8 (0.3–26.6)	20.3 (6.6–50.0)	NA
- Diarrhoea	1.7 (0.1–3.0)	5.9 (0.0–30.0)	6.5 (5.3–7.6)	4.7 (0.1–15.0)	5.6 (1.2–25.4)	6.5 (5.8–7.2)
- Nausea	2.6 (0.0–4.0)	6.8 (0.0–44.4)	5.0 (3.3–6.8)	5.2 (1.7–10.5)	7.9 (0.0–24.5)	8.1 (6.6–9.8)
General disorders, administration site condition	0.0	7.1 (2.1–14.4)	NA	0.9 (0.0–17.5)	8.1 (0.0–22.9)	NA
- Fatigue	NA	3.5 (0.0–10.9)	4.5	10.3	5.4 (1.5–14.1)	3.1
Hepatobiliary disorders	NA	3.8 (0.6–30.6)	NA	NA	5.2 (0.7–52.0)	NA
- Abnormal hepatic function	NA	4.5 (2.4–30.6)	NA	NA	4.9 (3.2–32.1)	NA
Infections and infestations	19.9 (3.0–36.8)	31.4 (4.5–63.0)	NA	33.0 (2.7–40.7)	36.2 (11.4–63.0)	NA
- Nasopharyngitis	1.9 (0.8–2.5)	7.2 (0.0–34.4)	10.3 (6.0–14.5)	4.8 (1.9–15.7)	8.3 (1.4–44.0)	11.7 (7.8–15.5)
- Sinusitis	13.0	3.9 (0.0–20.0)	3.8	10.4 (0.0–15.0)	5.5 (0.8–23.9)	6.2
- Upper respiratory tract infection	3.2 (0.2–9.0)	7.4 (0.0–24.1)	8.3 (7.5–9.1)	6.5 (0.1–30.0)	8.7 (1.2–34.0)	8.5 (5.8–11.2)
- Urinary tract infection	2.3 (2.1–2.5)	4.4 (0.0–21.0)	5.9 (5.3–6.5)	3.0 (0.0–25.0)	5.1 (0.9–26.5)	5.5 (2.7–8.3)
Injury, poisoning, procedural complications	0.8 (0.3–2.3)	6.0 (0.0–28.0)	0.8	2.5 (0.9–31.7)	8.8 (0.0–48.0)	3.1
Laboratory Investigation	3.8 (0.8–66.5)	2.7 (0.0–18.7)	NA	4.4 (2.7–41.0)	7.0 (1.1–23.2)	NA
- Raised ALT	6.6 (0.9–12.2)	2.8 (0.0–25.9)	5.4	5.0 (0.9–8.2)	3.7 (0.0–41.4)	11.9
Metabolism, nutrition disorders	NA	1.2 (0.0–5.5)	12.0	NA	3.3 (1.3–39.5)	12.3
- Hypercholesterolemia	NA	0.9(0.0–3.7)	12.0	NA	3.1 (0.0–8.8)	12.3
Musculoskeletal, connective tissue disorder	2.7	17.3 (12.6–30.3)	NA	8.7 (1.8–15.6)	13.3 (6.8–25.0)	NA
- Back pain	1.5 (1.4–1.6)	2.5 (0.0–11.3)	6.5 (5.3–7.6)	2.3 (1.0–8.4)	4.7 (0.0–15.5)	7.2 (4.0–10.5)
Nervous system disorders	0.0	8.7 (1.4–16.9)	NA	2.3 (0.3–4.2)	11.6 (6.5–19.0)	NA
- Headache	5.1 (1.9–23.0)	5.4 (0.0–25.9)	6.5 (5.3–7.6)	5.0 (0.0–16.0)	7.2 (1.5–26.0)	9.5 (6.5–12.4)
Respiratory, thoracic, mediastinal disorders	1.5	5.1 (0.0–20.3)	5.8	3.5 (0.0–8.8)	6.3 (0.5–54.1)	7.6
Ski/s.c. tissue disorder	0.4 (0.1–0.6)	2.9 (0.0–18.0)	2.4 (2.3–2.5)	4.2 (0.0–11.8)	6.3 (0.6–26.2)	3.9 (2.3–5.4)
- Rash	NA	1.8 (0.0–18.0)	2.4 (2.3–2.5)	5.0 (0.0–11.8)	6.5 (0.6–15.7)	3.9 (2.3–5.4)
Vascular disease	0.7 (0.0–2.3)	3.3 (0.0–12.5)	5.0 (3.0–6.9)	2.9 (0.5–7.5)	4.6 (0.0–22.0)	4.3 (3.9–4.7)
- Hypertension	0.7 (0.0–2.3)	3.0 (0.0–12.5)	5.0 (3.0–6.9)	2.9 (0.5–7.5)	4.4 (0.8–22.0)	4.3 (3.9–4.7)

Values given in median percentages (minimum–maximum). AE, adverse events; ALT, alanine amino transferase; DMARD, disease-modifying antirheumatic drug; csDMARD, conventional synthetic DMARD; bDMARD, biological DMARD; NA, not available; s.c., subcutaneous.

**Table 4:** Total (random/fixed effect) OR with 95% confidence intervals and *p* values, together with *p*-values of Egger’s tests. For ORs, *p* < 0.05 indicates a significant difference between prevalences in placebo and verum groups. With *p* < 0.05, Egger’s tests indicate a possible publication bias (highlighted in bold letters).

	Odds Ratios		*p*-Value for	
Adverse Event (AE)	OR	95% CI for OR	*p* Value for OR	Egger’s Test
Injection site erythema	3.98	2.66 to 5.95	<0.001	0.930
≥1AE	1.31	1.21 to 1.42	<0.001	0.162
SAE	1.17	1.08 to 1.27	<0.001	0.618
AE leading to withdrawal	1.39	1.19 to 1.61	<0.001	0.123
Death with AE	0.97	0.65 to 1.43	0.858	0.519
Blood and lymphatic system disorders	2.26	1.41 to 3.65	0.001	0.859
- Neutropenia	7.69	2.66 to 22.20	<0.001	0.670
Hypercholesterolaemia	1.28	1.28 to 1.40	<0.001	0.129
Infections and infestations	1.17	1.11 to 1.25	<0.001	0.574
- Sinusitis	1.47	1.29 to 1.69	<0.001	0.470
- Nasopharyngitis	1.30	1.19 to 1.42	<0.001	0.178
- Urinary tract infections	1.24	1.12 to 1.38	<0.001	0.416
- Upper respiratory tract infections	1.80	1.09 to 1.27	<0.001	0.371
Gastrointestinal disorders	1.41	1.12 to 1.77	0.004	0.623
- Nausea	1.00	0.86 to 1.17	0.985	**0.018**
- Diarrhoea	1.16	1.04 to 1.29	0.006	**0.022**
- Hepatobiliary disorders	1.08	0.85 to 1.36	0.542	0.274
- Abnormal hepatic function	1.09	0.83 to 1.44	0.537	0.400
Musculoskeletal and connective tissue disorders	0.75	0.64 to 0.88	<0.001	0.434
- Backpain	1.40	1.20 to 1.63	<0.001	0.623
- Aggravation of RA	0.59	0.48 to 0.73	<0.001	0.141
Respiratory, thoracic, mediastinal disorder	1.19	1.04 to 1.35	0.010	0.265
Skin and subcutaneous tissue disorders	1.78	1.56 to 2.03	<0.001	0.401
- Rash	1.57	1.33 to 1.86	<0.001	0.158
Vascular disorders	1.51	1.33 to 1.71	<0.001	0.260
- Hypertension	1.51	1.33 to 1.72	<0.001	0.094
General disorder, administration site condition	1.39	0.95 to 2.03	0.086	0.984
Abnormal laboratory parameters	2.01	1.33 to 3.04	0.001	0.098
- Raised ALT	1.59	1.19 to 2.13	0.002	0.132
Injury, poisoning and procedural complications	1.55	1.14 to 2.10	0.005	0.317
Serious infections and infestations	1.57	1.32 to 1.87	<0.001	**0.009**

SAE, severe adverse event; CI, confidence interval; OR, odds ratio.

## Data Availability

The authors provide the full dataset on request.

## Supplementary Materials

The following is available online at https://www.mdpi.com/article/10.3390/biom12020303/s1, Table S1: List of references for the 101 double-blind, placebo-controlled, randomized interventional trials in rheumatoid arthritis, selected for the meta-analysis of adverse events.
